# Remarks on the Black Vomit of Yellow Fever

**Published:** 1839-10-19

**Authors:** F. M. Robertson

**Affiliations:** Augusta, Ga.


### MEDICAL EXAMINER.

DEVOTED TO MEDICINE, SURGERY, AND THE COLLATERAL SCIENCES.

No. 42.] PHILADELPHIA, SATURDAY, OCTOBER 19, 1839. [Vol. II.

*Remarks on the Black Vomit of Yellow Fever.* By F. M. Robertson, M. D.

To the Editors of the Medical Examiner.

Gentlemen:—Should you deem the following observations, hastily thrown together during an unusual press of professional engagements, worthy of record, they are at your disposal.

A most destructive epidemic has prevailed in our city since the early part of August. Various opinions were, at first, entertained as to the nature of the disorder. The symptoms, course, and termination of the disease, in connection with the post mortem appearances, leave no doubt as to its being, unequivocally, "yellow fever," or what is styled in Charleston, S. C., "*strangers' fever.*" Some, who still contend that it is not yellow fever, assert that the black matter thrown from the stomach during the life of the patient, and found in it after death, is nothing more than black bile, mixed with the secretions of the stomach, the ordinary production of autumnal bilious fevers.

The late Dr. Physick, I think, was among the first to point out the remarkable difference between the matter of black vomit and the black bile. I repeated his experiments, at a post mortem examination, to-day. The subject of the examination had laboured under the usual symptoms of yellow fever, and expired throwing up large quantities of "black vomit." Upon exposing the cavity of the abdomen, the cardiac and pyloric extremities of the stomach were carefully tied, so that nothing could escape from, or enter into, that viscus: the gall-bladder was secured in the same manner, and both removed from the body. The stomach contained about a pint of black vomit, answering, in every particular, to the description given by different writers upon the subject. The gall-bladder, which was of the natural size, was two-thirds full of deep black bile, of nearly the consistence of tar.

I first spread the bile upon a white sheet of paper; it presented the usual dark green tinge, slightly inclining to yellow. The black vomit from the stomach was also treated in the same manner; the colour and appearance were totally different from that of the black bile. The fluid part of the black vomit resembled dirty coffee, while a thick dark sediment, resembling the coffee grounds, was deposited on the paper.

Some of the black bile was next placed in a vessel containing clear water; it was completely soluble, communicating its peculiar colour to the water. The black vomit was next placed in a similar quantity of pure water. After the most violent agitation, the dark floculi would sink to the bottom of the vessel, no portion being dissolved, and the water returning to its natural appearance. One other test was made; this experiment was tried, as we are informed, by Dr. S. Cooper; I am not at present aware of any other authentic instance. Knowing that the intense bitterness of the bile would be communicated to any substance, however slightly it might be impregnated with it, I resolved to try the different impressions that would be made upon the organs of taste, by the two substances. I first tasted the black bile, which was intensely bitter. After washing the mouth thoroughly from the impression produced by the bile, I then tasted the black vomit taken from the stomach. This communicated a slightly sour or subacid taste; with this exception, it was entirely insipid—not the slightest traces of bile were discoverable from the taste. This test was repeated by Dr. A. G. Howard, of Charleston, S. C., who assisted in the post mortem examination, and the same characters were recognised by him. The experiments were made in the presence of the Hon. A. Cumming, the indefatigable and public spirited mayor of our city, and Mr. A. Sibley, one of our most estimable citizens.

There are many other facts connected with the epidemic, which would not be unworthy of your notice. My numerous engagements render it impossible for me to say more at present. If such facts as these cannot convince the sceptical, argument would certainly be useless—worse than "water spilt upon a rock."

*Augusta, Ga.*, October 10, 1839.

